# Decreased Synaptic Vesicle Glycoprotein 2A Binding in the Human Postmortem Essential Tremor Cerebellum: Evidence of Reduction in Synaptic Density

**DOI:** 10.21203/rs.3.rs-2838184/v1

**Published:** 2023-05-02

**Authors:** Yanghong Yang, Chao Zheng, Baosheng Chen, Nora C. Hernandez, Phyllis L. Faust, Zhengxin Cai, Elan D. Louis, David Matuskey

**Affiliations:** Yale School of Medicine; Yale School of Medicine; Yale School of Medicine; University of Texas Southwestern School of Medicine; Columbia University Vagelos College of Physicians and Surgeons and the New York Presbyterian Hospital; Yale School of Medicine; University of Texas Southwestern School of Medicine; Yale School of Medicine

**Keywords:** Synaptic vesicle glycoprotein 2A, Autoradiography, Postmortem, Essential tremor, Cerebellum, [F18]SDM16

## Abstract

**Objective:**

Despite being one of the most prevalent neurological diseases, the pathophysiology of essential tremor (ET) is not fully understood. Neuropathological studies have identified numerous degenerative changes in the cerebellum of ET patients, however. These data align with considerable clinical and neurophysiological data linking ET to the cerebellum. While neuroimaging studies have variably shown mild atrophy in the cerebellum, marked atrophy is not a clear feature of the cerebellum in ET and that a search for a more suitable neuroimaging signature of neurodegeneration is in order. Postmortem studies in ET have examined different neuropathological alterations in the cerebellum, but as of yet have not focused on measures of generalized synaptic markers. This pilot study focuses on synaptic vesicle glycoprotein 2A (SV2A), a protein expressed in practically all synapses in the brain, as a measure of synaptic density in postmortem ET cases.

**Methods:**

The current study utilized autoradiography with the SV2A radioligand [^18^F]SDM-16 to assess synaptic density in the cerebellar cortex and dentate nucleus in three ET cases and three age-matched controls.

**Results:**

Using [^18^F]SDM-16, SV2A was 53% and 46% lower in the cerebellar cortex and dentate nucleus, respectively, in ET cases compared to age-matched controls.

**Conclusion:**

For the first time, using *in vitro* SV2A autoradiography, we have observed significantly lower synaptic density in the cerebellar cortex and dentate nucleus of ET cases. Future research could focus on *in vivo* imaging in ET to explore whether SV2A imaging could serve as a much-needed disease biomarker.

## Introduction

Essential tremor (ET) is among the most prevalent neurological diseases. An estimated 7 million individuals in the United States are affected with ET, representing approximately 2.2% of the entire US population [38]. The prevalence among individuals aged 65 and older reaches approximately 6–8%, and this value rises to 20% or higher among individuals in their 90s [37]. Given this high prevalence, ET patients are ubiquitous, deriving their care broadly from a variety of providers, including general practitioners, neurologists, and movement disorders neurologists [54]. The medical costs associated with ET are not trivial; a recent analysis predicted that, across the population, aggregated additional spending attributable to ET among Medicare beneficiaries was between $1.5 billion and $5.4 billion each year [26]. The pathophysiology of ET is not fully understood. However, over the past 10 to 20 years, an intensive effort to bank ET brains has resulted in postmortem studies involving more than 200 brains [33]. From these studies, one sees evidence of an emerging underlying neuropathology [34]. Postmortem changes are centered in and around Purkinje cells (PCs), with numerous studies demonstrating PC loss [16, 32, 36]. These data align well with considerable clinical and neuroimaging data that have linked ET to the cerebellum [5, 15, 19, 24, 29–31, 42] and which suggest that ET is neurodegenerative in nature [4, 6, 7, 12, 18, 22, 23, 27, 28, 40, 41, 47, 50–52]. While neuroimaging studies have variably shown mild atrophy in the cerebellum or specific cerebellar folia [2, 13, 17, 49], or no detectable volumetric changes [10], the sense is that marked atrophy is not a clear feature of the ET cerebellum and that a search for a more suitable neuroimaging signature of neurodegeneration is in order.

Recently, synaptic vesicle glycoprotein 2A (SV2A), a protein expressed in practically all synapses in brain [3, 9], has been developed as a marker for clinical neuroimaging studies [11, 14, 20]. Previous work has demonstrated a strong association between SV2A and the gold standard synaptic density marker, synaptophysin, in western blot and confocal microscopy [21], showing that SV2A has promise for *ex vivo* and *in vivo* characterizations of synapses. This has already been investigated in several neurological disorders, such as Parkinson’s and Alzheimer’s disease [43–45], as several positron emission tomography (PET) radioligands have been developed and validated in preclinical and clinical populations [14, 48, 55, 56]. In this pilot study, we investigate the synaptic differences in the postmortem cerebellum of ET cases versus controls by using the SV2A radioligand [^18^F]SDM-16 with high binding affinity and metabolic stability [55, 56].

## Methods

### Study Subjects and Clinical Assessment

3 ET brains and 3 control brains (Table 1) were obtained through the Essential Tremor Centralized Brain Repository (ETCBR) in the New York Brain Bank. ET diagnoses were carefully assigned by a senior movement disorders neurologist (E.D.L.) utilizing three sequential methods [1]. Briefly, the clinical diagnosis of ET was initially assigned by treating neurologists, and second, confirmed by E.D.L. using questionnaires, review of medical records and review of Archimedes spirals. Third, a detailed, videotaped, neurological examination was performed, and action tremor was rated, and a total tremor score assigned [range = 0–36 (maximum)]. Combined with the questionnaire data, the final diagnosis of each ET case was revisited, using previously published diagnostic criteria, which have been shown to be both reliable and valid [39]. None of the ET cases had a history of traumatic brain injury, exposure to medications with associated cerebellar toxicity (e.g., chemotherapeutic agents), or heavy ethanol use, as previously defined [1, 25]. Every 6 months, a follow-up semi-structured telephone evaluation was performed, and hand-drawn spirals were collected; if there was concern about a new, emerging movement disorder, a detailed, videotaped, neurological examination was repeated. The three control brains, from the New York Brain Bank, were clinically free of ET and other neurodegenerative disorders, including Alzheimer’s disease, Parkinson’s disease, or progressive supranuclear palsy [35].

## Postmortem Methods

Brains had a complete neuropathological assessment, including standardized measurements of brain weight (in grams) and postmortem interval (PMI, hours between death and placement of brain in a cold room or upon ice). Braak and Braak Alzheimer’s disease staging for neurofibrillary tangles and CERAD (The Consortium to Establish a Registry for Alzheimer’s Disease) stage for neuritic plaques were assigned [8, 46].

## Autoradiography Methods

**Figure 1:**
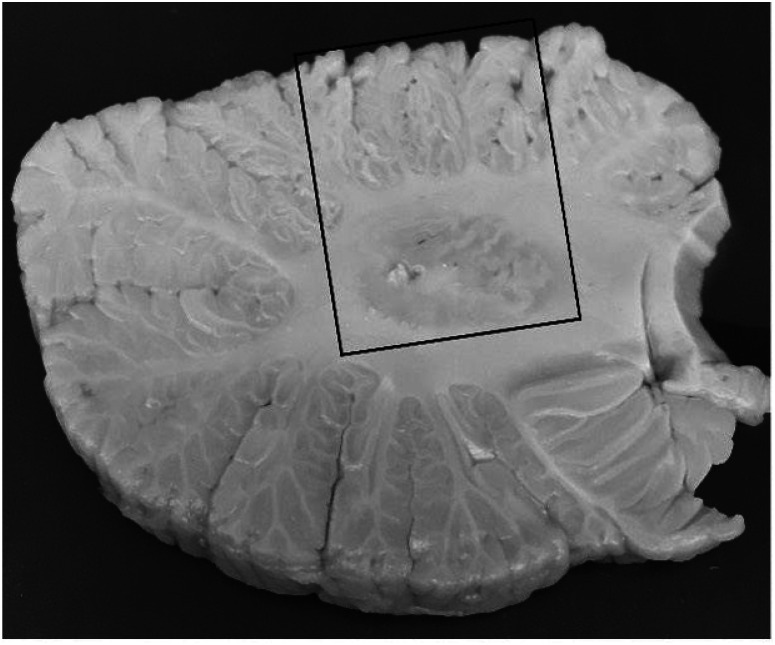
Cerebellum block 14 (V-VI): A standard 3 × 20 × 25 mm cerebellar tissue block (outlined in rectangle) was fresh frozen from a parasagittal slice located 1–1.5 cm from the cerebellar midline and containing posterior quadrangulate lobules and the underlying dentate nucleus.

We obtained a standard 3 × 20 × 25-mm fresh frozen tissue block from each brain from a parasagittal slice located 1–1.5 cm from the cerebellar midline and containing anterior and posterior quadrangulate lobules and the underlying dentate nucleus (Fig. 1). 14-um thick cryostat sections were prepared from portions of the frozen human cerebellar cortex and dentate nucleus and placed on Superfrost^™^ Plus slides (Fisher Scientific). Slides with frozen sections were thawed on ice for 10 minutes prior to the equilibration in an aqueous incubation buffer (30 mM HEPES, 0.56 mM MgCl_2_, 110 mM NaCl, 5 mM KCl, 3.3 mM CaCl_2_, 0.1% fatty acid free bovine serum albumin and pH 7.4) for 10 min. Half of the slides were incubated with the radioligand only while the other half were incubated with unlabeled SDM-16 (2 μM) for 10 mins prior to the administration of the radioligand, [^18^F]SDM-16, at 1.5–2 μCi/ml for 45 mins to block the radioligand and verify that binding was specific. Afterwards, the slices were washed three times (20 seconds each) using bovine-serum albumin-free incubation buffer and twice in distilled water (5 seconds each). The tissues were blow dried (20 mins) and exposed to a phosphor imager plate (Fuji) for 12 hours. The plates were scanned using Typhoon 5 Phosphor Trio imaging system, and the resulting images were quantified using Image J (Fiji) by measuring the signal intensity of the whole tissue area.

The blocking results with unlabeled SDM-16 blocked 96.2% and 92.1% of SV2A signals in cerebellar cortex and dentate nucleus respectively, which indicated high levels of specific binding for SV2A of the radioligand [^18^F]SDM-16 in the human cerebellum.

## Statistical Analyses

Statistical analyses were performed in SPSS (version 28.0). Kolmogorov-Smirnov tests indicated that all continuous variables were normally distributed. Student’s independent samples t-test and chi-square tests were used when comparing two groups (ET cases vs. controls).

## Results

### Demographic and Primary Postmortem Features of ET Cases and Controls

The 3 ET cases and 3 controls were demographically matched for age at death, brain weight, Braak and Braak stage and CERAD neuritic plaque stage (Table 1). The postmortem interval was non-significantly longer in ET cases than controls. No cases were heavy ethanol users, and none had lifetime exposure to cerebellar toxic medications.

## Autoradiographic Studies

**Figure 2:**
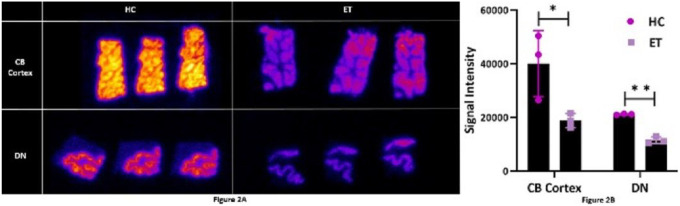
**2A** Autoradiograms with binding of [^18^F]SDM-16 to synaptic vesicle glycoprotein 2A in the cerebellar cortex (CB cortex) and dentate nucleus (DN) of HC and ET brains. **2B** [^18^F]SDM-16 signal intensity in CB cortex and DN in HC and ET brains (Student’s t tests, *p<0.05, **p<0.01).

Representative autoradiography slides of [^18^F]SDM-16 to SV2A in the cerebellar cortex and dentate in ET and controls are displayed in Fig. 2A. The [^18^F]SDM-16 ligand showed high binding signal in gray matter regions and low uptake in white matter, as would be expected for a synaptic protein. [^18^F]SDM-16 displayed significantly lower uptake in ET brains in both the cerebellar cortex (−53%) and dentate (−46%) in comparison with control brains (Figs. 2A and 2B).

## Discussion

Using a generalized marker for synaptic density in the postmortem human cerebellum, we present evidence of lower cerebellar synaptic density in ET. Using [^18^F]SDM-16, we observed that SV2A uptake was 53% and 46% lower in the cerebellar cortex and dentate nucleus, respectively, in ET compared to age-matched controls. These data are consistent with larger data that both links ET to the cerebellum [5, 15, 19, 24, 29–31, 42] and that indicates changes that are likely degenerative [4, 6, 7, 12, 18, 22, 23, 27, 28, 40, 41, 47, 50–52].

Our focus on the cerebellar cortex in these studies was based on a wealth of prior postmortem data demonstrating changes in the cerebellar region in ET [33]. The dentate nucleus is also considered to be critical in the pathophysiology of ET, as it is a prime relay point for descending Purkinje cell output, and prior studies have shown evidence of changes in this critical cerebellar nucleus [32, 53]. Taken together, SV2A could be a marker for synaptic and neuronal loss, showing degeneration in these brain regions.

The study was not without limitations. First, the number of subjects was small (N = 3 ET and 3 controls). However, even with this small sample size, significant reductions in SV2A were detected both in the cerebellar cortex and dentate nucleus in ET, and across all samples studied, with no overlap in distribution of data points in ET and controls. A larger study is now warranted. Second, *in vivo* studies across the spectrum of the disease could provide more detailed evidence of possible synaptic decreases at different stages of disease and with symptomology, something now possible with SV2A imaging.

In conclusion, we observed significantly lower synaptic density in the cerebellar cortex and dentate nucleus in ET compared to controls. Future research on synaptic density could focus on *in vivo* imaging in ET, exploring whether SV2A imaging could serve as a much-needed disease biomarker.

## Figures and Tables

**Table 1:** Clinical and pathological data in essential tremor cases and controls.

	Control (n = 3)	ET (n = 3)	Significance
Age at death (years)	88.3 (4.0)	86.3 (3.5)	0.55^a^
Gender			0.08^b^
Male	3 (100%)	1 (33.3%)	
Female	0 (0%)	2 (66.7%)	
Post-mortem interval (hours)	12.9 (12.3)	20.6 (9.9)	0.45^a^
Brain weight (grams)	1212.3 (214.0)	1183.5 (69.7)	0.84^a^
Braak and Braak neuronal tangle stage			0.99^b^
IV	2 (66.7%)	2 (66.7%)	
V	1 (33.3%)	1 (33.3%)	
CERAD^ neuritic plaques score			0.37^b^
0	2 (66.7%)	2 (66.7%)	
1	0 (0%)	1 (33.3%)	
2	1 (33.3%)	0 (0%)	

Values represent mean ± standard deviation or number (percentage).

a Student’s independent sample t-test.

b Chi-square test.

^ The Consortium to Establish a Registry for Alzheimer’s Disease

## Data Availability

All data can be accessed with reasonable requests.
